# Recent outbreak of diphtheria in Pakistan; short communication

**DOI:** 10.1097/MS9.0000000000000832

**Published:** 2023-05-10

**Authors:** Syeda Ilsa Aaqil, Sidhant Ochani, Md. Al Hasibuzzaman

**Affiliations:** a Department of Medicine, Jinnah Sindh Medical University, Karachi; b Department of Medicine, Khairpur Medical College, Khairpur Mir’s, Pakistan; c Institute of Nutrition and Food Science, University of Dhaka, Dhaka, Bangladesh

**Keywords:** Diphtheria; *Corynebacterium diphtheria*; outbreak; Pakistan; Diphtheria and Tetanus Toxoids and Pertussis Vaccine

## Abstract

Diphtheria, a vaccine-preventable bacterial infection caused by *Corynebacterium diphtheria* usually starts with sore throat and fever and often results in breathing difficulties, heart rhythm problems, and rarely membranous pharyngitis. Although nursing these complications can help most people survive diphtheria, but it can be deadly in 5–10% of cases with higher death rates observed in children under 5 years of age or adults above 40. For the year 2022, 92 cases have been reported by seven European countries. Sixty-six of the reported cases presented with cutaneous diphtheria caused by *Corynebacterium diphtheria* while cases of respiratory diphtheria have also been reported, including one fatal case. The increase in diphtheria cases can be linked to an increased volume of migrants from diphtheria-endemic countries causing transmission of pathogens from countries of origin to recipient countries. Today the authors can treat diphtheria infections by using antibiotics and also prevent the disease with a vaccine. General population should be given awareness and educated in regard to disease prevention and appropriately implement administration of Diphtheria and Tetanus Toxoids and Pertussis Vaccines among people at risk for their own protection and urgently call for an action to eliminate the disease before its further spread as an outbreak.

Diphtheria, a vaccine-preventable bacterial infection caused by *Corynebacterium diphtheria* resulted in 8639 cases in 2021 globally with Ethiopia accounting for 4453 that is 51.55% of the world’s diphtheria cases. At the same time, 92.42% of total cases are reported from India, Yemen, Indonesia, and Pakistan^1^. Two primary determinants that is (1) the ability of a given strain of *C. diphtheriae* to colonize in the nasopharyngeal cavity and/or on the skin, and (2) its ability to produce diphtheria toxin are the foundation of the pathogenesis of diphtheria^2^. Diphtheria produces a very strong toxin that can damage organs and may lead to death. It usually causes inflammation on mucosal surfaces of the upper respiratory tract and systemic lesions on the heart and nerves. This can lead to a sore throat and fever and often results in breathing difficulties, heart rhythm problems, and rarely membranous pharyngitis^3^. Although nursing these complications can help most people survive diphtheria, it can be deadly in 5–10% of cases with higher death rates observed in children under 5 years of age or adults above 40. Diphtheria infections can also be lethal to a pregnant woman and can result in the loss or premature birth of a baby^4^. As far as transmission of diphtheria is concerned, it can occur via inhalation of airborne droplets released during coughing or sneezing, or handling contaminated personal or household items. People sharing the same roof, people with a history of frequent, close contact with the patient, and people exposed to secretions from infected sites are more prone to diphtheria infections^5^.

For the year 2022, 92 cases have been reported by seven European countries. Sixty-six of the reported cases presented with cutaneous diphtheria caused by *C. diphtheria* while cases of respiratory diphtheria have also been reported, including one fatal case. The increase in diphtheria cases can be linked to an increased volume of migrants from diphtheria-endemic countries causing transmission of pathogens from countries of origin to recipient countries^6^.

Pakistan is currently facing a diphtheria outbreak. Although diphtheria cases in Pakistan fluctuated substantially in recent years, they increased through the 2002–2021 period ending at 169 in the year 2021^7^, and a total of 45 fatalities of children and teenagers in 2022. In Pakistan, the poor quality of the vaccination regimen and unavailability of anti-diphtheria serum led to reporting of 46 cases of diphtheria across Khyber Pakhtunkhwa, and the numbers are expected to increase. Out of 46 cases, 17 are from Peshawar; 9 from Karak; 9 from Laki Marwat, 4 from Mardan; and 1 each from Charsadda, Hangu, Buner, Swabi, Shangla, Mansehra, and Malakand. Moreover, hundreds of cases are suspected to be reported from Sindh, Balochistan, Punjab, and Khyber Pakhtunkhwa as well as Azad Kashmir and Gilgit-Baltistan. Following the deaths of dozens of children throughout the country due to diphtheria, the National Institute of Health (NIH) Islamabad has ‘advised’ the Drug Regulatory Authority of Pakistan (DRAP) to ensure the availability of Diphtheria Antitoxin in the country, which is used to treat infected patients along with antibiotics^8,9^.

Before antibiotics were available, diphtheria was a common illness in young children. But today we can treat diphtheria infections by using antibiotics and also prevent the disease with a vaccine. This can be especially beneficial against diphtheria infections in the respiratory system, skin, and other parts of the body (e.g. eyes, blood). Patients no longer infect others 48 h after they begin taking antibiotics although, completion of the antibiotic course is necessary for the total removal of bacteria. The diphtheria vaccine is usually combined with vaccines for tetanus and whooping cough (pertussis). The three-in-one vaccine is known as diphtheria, tetanus, and pertussis vaccine^4^. Diphtheria antitoxin is mainly recommended for respiratory diphtheria infections, but it is rarely used for diphtheria skin infections. Oral Penicillin/Erythromycin/Azithromycin can also be given to prevent diphtheria infection. The general population should be educated about preventing disease and getting vaccinated. People at high risk or those living in areas with an outbreak, or are pregnant should be informed via automated messages services or caller tune campaigns. An urgent call for action to eliminate the disease before its further spread as an outbreak is needed.

## Ethical approval

Not applicable.

## Consent

Not applicable.

## Source of funding

The author(s) received no financial support for the research, authorship, and/or publication of this article.

## Author contributions

Literature review and manuscript was written by S.I.A. Review editing was done by M.A.H. and S.O., formatting and referencing was done by S.O.

## Conflicts of interest disclosure

The author(s) declared no potential conflicts of interest concerning the research, authorship, and/or publication of this article.

## Research registration unique identifying number (UIN)

Not applicable.

## Guarantor

Sidhant Ochani.

## Data availability statement

Not applicable.

## Provenance and peer review

Not commissioned, externally peer-reviewed.
